# Preventive measures aimed at minimizing the risk of African swine fever virus spread in pig farming systems

**DOI:** 10.1186/s13028-016-0264-x

**Published:** 2016-11-29

**Authors:** Silvia Bellini, Domenico Rutili, Vittorio Guberti

**Affiliations:** 1 Istituto Zooprofilattico Sperimentale della Lombardia ed Emilia Romagna (IZSLER), Brescia, Italy; 2 Private Consultant, Perugia, Italy; 3 Istituto Superiore per la Protezione e la Ricerca Ambientale (ISPRA), Ozzano Emilia, Bologna, Italy

**Keywords:** African swine fever, ASF, Domestic pigs, Wild boar, Wildlife, Epidemiology, Prevention, Bio-security

## Abstract

African swine fever (ASF) is one of the most severe diseases of pigs; it has a drastic impact on the pig industry, causing serious socio-economic consequences to pig farmers and pork producers. In Europe, there are currently two main clusters of infection; one in Sardinia caused by strains of African swine fever virus (ASFV) belonging to genotype I and another in Eastern Europe caused by strains of ASFV belonging to genotype II. The latter is inducing an acute form of ASF and it represents a serious threat to the pig sector. ASF is a disease for which there is no effective vaccine; therefore, prevention has a pivotal role in the control strategy of the disease. This review describes the main preventive measures to adopt to mitigate the risk of ASF spread in pig farming systems.

## Background

African swine fever (ASF) is a contagious and fatal disease of pigs caused by African swine fever virus (ASFV) currently classified as the only member of the *Asfarviridae* family. ASF is one of the most serious diseases of pigs; it can severely affect and disrupt regional and international trade with animals and animal products with a serious socio-economic impact on pig farming. The disease is mainly transmitted by direct contact between infected and susceptible pigs or through the ingestion of ASFV contaminated pork products [1, 2]. Wild boar (*Sus scrofa*) and feral pigs in general are also susceptible to ASFV and they show similar clinical signs and mortality rates as domestic pigs [3]. In certain areas, soft ticks of *Ornithodoros* genus can be involved in the epidemiology of the disease.

African swine fever is endemic in sub-Saharan Africa and on the Italian island of Sardinia. In 2007, the disease was reported in Georgia, most probably originating from Southeast Africa. From Georgia, the virus spread to Armenia and to Azerbaijan, meanwhile it crossed the Caucasus Mountains spreading into several states of the Russian Federation where, in certain areas, the disease became endemic representing a constant threat for the neighbouring countries [3]. In 2013, from the Russian Federation, the virus reached Belarus and Ukraine and later, in 2014, it was introduced into Lithuania, Poland, Latvia and Estonia affecting both domestic pigs and wild boar. Based on the characteristics of the virus and on the epidemiological findings, the introduction into the Baltic countries and into Poland was most probably from Belarus [4]. The ASFV strain that is currently circulating in the Eastern European countries and Baltic States is a highly virulent and highly lethal strain, belonging to genotype II which has 100% sequence homology with the ASFV identified in Belarus in June 2013 [5].

More than 1 year after its introduction into Poland and into the Baltic countries, ASF is still spreading in wild boar in the eastern member states of the European Union (EU) with few spill overs into the domestic pig population, while in Sardinia, Ukraine and in the Russian Federation the virus continues to spread in both wild boar and domestic pigs. The current epidemiological situation in the Eastern part of Europe represents a constant threat to the EU livestock sector, particularly if the infection pressure remains high at the eastern borders of the EU. The presence of ASF in Sardinia seems to pose a limited risk to the pig sector of the ASFV free territories of the EU. Indeed, in 38 years ASFV spread from the island of Sardinia only on one isolated occasion [6]. No vaccine or drugs are available to prevent ASFV infection. Therefore, it is particularly important to prevent the introduction of the disease in free areas and to reduce, as much as possible, the persistence of the virus in the infected ones.

Prevention and early detection play a key role in the control strategy for ASF and enhancing early detection would also improve the efficacy of the disease control measures. In the EU, the provisions to apply in case of infection are mainly targeted towards domestic pigs and are based on surveillance, epidemiological investigations, tracing of pigs and stamping out infected holdings [7]. However, in order to mitigate the risk of spread, such measures are to be applied in combination with strict preventive measures in domestic pig holdings. The basic elements of biosecurity derive from the knowledge of the epidemiology of the disease, the duration of pathogen excretion in infected animals, the main routes of excretion, survival of the pathogen in the environment and its routes of infection. Some basic principles of biosecurity apply to all farming systems and all diseases [8]. However, in order to better address preventive and control measures, the main practical biosecurity measures need to be tailored to the targeted disease and to the farming systems in which they are to be implemented [8]. Indeed, biosecurity measures are normally quite standardized in commercial holdings, whilst they are not well defined and of easy implementation in the backyard sector.

The aim of this paper is to define the main preventive measures to adopt to minimize the risk of ASF spread in the pig sector, including both industrial and backyard production systems. Indeed, backyard holdings with poor biosecurity in place are currently playing an important role in the maintenance and spread of ASFV in the infected eastern neighbouring countries of the EU [3]. Moreover, considering the significant involvement of wild boar in the current epidemic in the Eastern parts of Europe, we also take into account the role played by wild boar at the interface with domestic pigs and the measures to adopt to properly manage the hunting activities.

## Search strategy

This critical review is based on a search in PubMed (http://www.ncbi.nlm.nih.gov/pubmed) using the terms African swine fever, ASF, domestic pigs, wild boar, wildlife, epidemiology, prevention and bio-security. Only articles in English were included in the text. EFSA Scientific Review and Opinions on ASF were used as source of additional information. Our experiences on ASF management and on the biological aspects of the disease were used to critically evaluate the articles and to identify the main preventive measures to be adopted against ASF. The EU legislation and OIE standards on ASF were consulted to ensure coherence with the proposed measures.

## Review

### African swine fever epidemiology

A range of wild and domestic pig species are susceptible to ASFV and different tick vector species, all belonging to *Ornithodoros* genus, can be involved in the epidemiology of the disease. Transmission and maintenance of ASF can occur in a sylvatic cycle and/or in a domestic pig cycle. Depending on the presence or absence of wild *Suidae* and arthropod vectors and on the type of pig production system, the epidemiology of the disease varies substantially between different habitats and regions. The African scenario includes the presence of ASFV-infected ticks, different species of wild suids acting as reservoirs for the disease, domestic pigs that have been exposed to ASFV for many years and partly ASF-resistant [9, 10]. These conditions do not exist in any European Country.

For the purpose of this review, we refer mainly to the epidemiological situation in Europe and to the domestic pig cycle. In Europe there are currently two main clusters of ASFV infection. One of them is in Sardinia where the disease was introduced in 1978, most probably from Iberian Peninsula [11], and it is caused by strains of ASFV belonging to genotype I [6]. The second is occurring in the eastern part of Europe and it is caused by strains of ASFV belonging to genotype II that has 100% sequence homology with the ASFV identified in Belarus in June 2013, a genetic variant of the virus introduced in Georgia in 2007 [5]. The latter is a highly virulent strain inducing an acute form of ASF that results in a mortality rate of 94.5–100% in both wild boar and domestic pigs [4, 12–15].

ASFV has a remarkable ability to survive for long periods in a protein rich environment and it remains stable at pH 4–10 [16]. The virus is not affected by meat maturation processes and meat from pigs slaughtered in the infective stages of ASF or that die spontaneously of the disease provides a good source of virus. ASFV persist in tissues for up to 6 months and can be infectious in susceptible animals fed with such meat [17]. The virus can also remain viable in the lymph nodes of the few survivors to the disease [18, 19] and, in such instance, the natural death of these animals can re-initiate a new epidemic cycle of ASF, if carcasses are not properly disposed and the virus gets in contact with a susceptible animal.

The virus is quite resistant to high temperatures and it requires exposure to a temperature of 60 °C for at least 20 min for inactivation [1]. Fresh and frozen pork, as well as smoked, salted and dried pork may contain infective quantities of ASFV [20, 21] whereas the virus is inactivated in heat-treated products [1]. Commercial processed products (such as ham or cured pork loin) contain no active virus 140 days after processing of the fresh meat started. ASFV is also able to survive for long periods in some tissues such as bone marrow, in spite of putrefaction [10]. This probably plays a role in infecting scavenging pigs in areas where the disease is endemic. The resistance of the virus to inactivation also means that transmission is possible via fomites such as contaminated clothing and shoes, equipment and vehicles.

Pigs are infected mainly through the oro-nasal route after contact with infected pigs or after feeding on virus-containing pork or contaminated products. All excretions and secretions of infected pigs such as blood, faeces, urine or saliva can contain virus, and ASFV may remain viable in blood and tissues for long periods [3, 5, 17, 19]. The onset of viremia is observed from 3 to 5 days post infection (DPI) and transmission via direct contact can occur for several weeks [22]. As regards ASFV genotype II, in pigs experimentally infected, it has been observed that the excretion of the virus started during the incubation period and lasted for the entire period of the disease. Hence, pigs were infectious from 4 to 10 DPI [23].

So far, with the strain circulating in the Baltic countries and in Poland there is no evidence that recovered pigs can become long-term carriers of the ASFV [2–4]. However, in large pig populations, ASF can be maintained for long periods in the area due to constant supply of susceptible pigs [10], illegal movements of infected pork meat, poor biosecurity in pig holdings, and aggregation of wild boar promoted by feeding [3].

The dynamic of the infection in the wild boar shows the same pattern observed in domestic pigs [24]. The role played by wild boar in the epidemiology of ASF is mainly determined by wild boar abundance and the geographical continuity of suitable habitats [25]. Indeed, it appears that in Sardinia wild boar have a limited role in the spread of the disease [26] whilst in the Baltic countries and Poland they are acting as reservoir [3, 4, 25].

Soft ticks of *Ornithodoros* species, in certain epidemiological contexts, can play a role in transmitting ASFV [19]. In Europe, only ticks of the *O. erraticus* complex have been reported in some countries around the Mediterranean Basin [27]. Ticks of the genus *Ornithodoros* are common in pig pens in many areas of Africa and in certain parts of the Iberian Peninsula, while the information available suggests that they are absent from Sardinia [28]. Knowledge on the occurrence of these ticks in other areas of Europe is rather incomplete. However, in the Baltic countries, in Poland and in Central Europe their presence seems unlikely, as their involvement in the epidemiological cycle of the disease [29]. Such ticks are able to retain ASFV for a long time, at least 5 years, and to transmit it to susceptible species [30, 31]. In addition, some of these ticks can transmit the virus from tick to tick through trans-stadial and trans-ovarian transmission [31]. *Ornithodoros* ticks mainly feed on animal species living in burrows or they are harboured inside pigsties in old buildings, where they hide in cracks and surfaces that provide sufficient humidity. Pigs are mostly accidental hosts, from which the ticks can be infected. Wild boar has never been found infested by *Ornithodoros* ticks, since wild boar normally rest on the surface, rarely on the same spot and never inside burrows. Therefore, such ticks can play an important role in maintaining local foci of infection, but not in the geographical spread of the virus [27].

### ASF risk factors, pathways of transmission and preventive measures

#### Risk factors for the spreading of ASFV

Few analytical studies have been carried out to identify risk factors for ASF at farm level. Factors found to increase the risk of outbreak, include free range pig management system [11], the previous occurrence of a disease in the farm [32], the presence of an infected pig farm in the neighbourhood or of an abattoir in the community, and visits by veterinarians and para-veterinarians [33]. A spatial regression analysis found density of the road network, of water bodies and of the domestic swine population to be associated with outbreaks in Russia [34] and a spatial spread model found the movement of infected animals to be the most important factor in the spread of ASFV [35]. In the backyard sector the main risk factors are human induced, such as illegal movements of infected pork meat and swill feeding together with suspected cases underreporting and “emergency sales” [36]. The backyard sector commonly uses swill as supplementary feed, which may include untreated ASFV contaminated pork or pig products. Often, the contaminated meat may have been stored chilled, frozen or after treatment and kept over long periods, thus acting as the main mechanism for ASFV maintenance and re-introduction. Recent epidemiological investigations carried out in Lithuania and Latvia have suggested that fresh grass and seeds contaminated by secretions from infectious wild boar could be possible source of infection for backyards [2]. Virus re-introduction and amplification mainly takes place in the backyard pigs and then ASFV seasonally spills over first to small farms and then to commercial pig farms. Additionally, the interaction between wild boar and domestic pigs can prolong ASFV circulation in both swine populations, as observed in many outbreaks in Sardinia and in the Russian Federation [26, 37]. In Sardinia, the number of backyards together with the illegal free ranging of pigs is considered a main risk practice for the persistence of ASF in certain areas of the island [6]. Indeed, free-range pigs grazing on vast municipal owned lands have been strongly associated with the maintenance of ASFV infection in the endemic area of Nuoro, as demonstrated by the intense viral circulation amongst such pigs [11]. In a recent study carried out in Sardinia, also the number of closed farms, roads density and the mean altitude have been associated with an increased risk of ASFV introduction in pig holdings [38].

In the Baltic countries and in Poland, it was observed that wild boar habitat suitability, neighbouring distance from infected wild boar and domestic pigs were the main risk factors for the introduction into EU free areas through infected wild boar [39]. Different studies highlighted the continuity of the geographical distribution of wild boar, wild boar management (hunting system, winter feeding), local density and size of the infected population, direct contact with dead infected wild boar, as the most relevant risks for the spread and the persistence of the virus in wild boar populations [4, 25, 40].

#### ASF pathways of transmission and preventive measures

Preventive measures to apply to mitigate the risk of spread of a disease in the pig production system are to be targeted to the main potential routes of transmission of the pathogen. Some of them are applicable across all production systems, others are not [8].

Based on the epidemiological characteristics of ASF, the disease pathways of transmission [19] are:

- Direct pig-to-pig contact.
- Consumption of contaminated feed (swill feeding).
- Vehicles and other fomites, clothing, footwear, surgical equipment.
- Workers and visitors.
- Slurry.
- Genetic materials.
- Bites from ticks.

##### Direct pig-to-pig contact

African swine fever virus is primarily transmitted by direct contact between infected and susceptible pigs. The spread of the virus from infected animals can start from the second DPI by means of saliva, ocular secretions and nasal discharges [17]. After a few days, the virus is shed via urine, faeces and semen. Aerosol can also transmit the virus over short distance [17, 22]. It is worth to be taken into consideration that infected pigs are most contagious during the incubation period of the disease, when they may shed virus for up to 48 h before showing clinical signs and during the clinical stage of disease, when a large amounts of virus is present in blood, secretions and excretions [23]. Given the characteristics of resistance of the virus, this pathway of transmission is extremely efficient both, locally (at farm level, village, and pasture) and long distance [4]. Therefore, in risk areas it is necessary to adopt specific measures to minimize the risk of introducing the disease into the herd by direct contact between infected and susceptible animals.

- Physical isolation of the herd
  It is aimed at limiting the potential opportunities for a susceptible animal to get physically in contact with an infected one. Indeed, if a pathogen does not enter the holding no infection can take place. Depending on the pig production system, the local geographic and socio-economic conditions and the capacity to invest resources, isolation of the holding can be obtained by maintaining adequate distances between farms, by full fencing the herd and installing a closed entrance to the farm area [8]. There are simple measures such as permanent housing of pigs and closed entrance to the premise that achieve the same objective and can be implemented also in rural villages with very limited resources. For the holdings of new settlement, physical location of the premise should be carefully planned in the light of maintaining adequate distances from neighbouring farms, slaughterhouses, meat-processing establishments, animal markets and frequently used roads [8].

- Introduction of new pigs into the herd
  No pigs should enter or leave the holding unless necessary and when necessary, adequate preventive measures shall be adopted to mitigate the risk. Pigs shall be introduced into the herd only from trusted and certified sources. To mitigate the risk of spread of pathogens, special attention shall be given to the management of animal transport and to the cleansing and disinfection procedures of the vehicles and the loading/unloading area. The number of suppliers for replacement stock should be limited and their health status carefully evaluated before purchase. Newly purchased pigs shall be maintained for a minimum of 30 days in quarantine or, at least, physically isolated from the rest of the herd. Frequency of introduction shall be limited too [8]. During the quarantine period the animals should be carefully checked to early detect the presence of conditions to avoid the introduction of diseased animals onto the herd. Clinical surveillance, is the most effective tool for early detection of ASF [4]. However, given the clinical similarity with other diseases of pigs, clinical surveillance should be supplemented, as appropriate, by serological and virological surveillance [41].

- Movement stand still
  When pigs are for breeding and production and they are destined for another holding, pigs must have remained in the holding of origin for a period of 30 days prior to loading or since birth if less than 30 days old [42].

- Pig markets
  In certain areas, live pig markets are extremely important for local trade. They are obvious mixing points and a potential source of disease. Pigs are brought to the market by owners or traders and they are a cross point where small and commercial producers, traders and butchers meet. Therefore, they are a crucial point for the spread of diseases. Once pigs are brought to the market, they comingle with other animals and they do not have any more the health status of the original herd. Therefore, to limit the risk of spread, pigs that have not been sold at market shall not be reintroduced into the original herd, unless they do not pass through an appropriate period of quarantine [8]. Pig markets should be kept under strict veterinary supervision and the animals are to be admitted to the market only if accompanied by a certificate attesting the favourable health status. However, in case ASF is confirmed in the area, live pig markets should be closed.

- Disposal of carcasses
  Vehicles collecting dead animals represent a major risk for the transmission of the disease. They shall not enter into the farm and pig carcasses are to be collected outside the fence. Drivers have to strictly follow farm biosecurity protocols and they should not enter the holding. Carcasses of domestic pigs and wild boar found dead in the infected areas shall be processed under official supervision and they should be checked and tested to early detect the presence of ASFV. Carcasses, discarded parts from slaughtered pigs shall be disposed by incineration or burial in an authorized land fill. No part of any wild boar, whether shot or found dead, shall be brought into a pig holding.

- Natural mating
  Natural mating provided by an external boar would also imply moving the boar or the sow for mating from one location to another. Such movements of pigs between herds are to be regarded as a dangerous practice. Therefore, in areas at risk such practice should not be conducted [8].

##### Consumption of contaminated feed (swill feeding)

Feeding of catering waste is a high risk practice as several diseases, including ASF, can be introduced in a healthy population through such feeding. It is documented that the majority of the outbreaks occurred in ASFV-free zones were the result of feeding food waste products from infected pigs to susceptible animals [16, 19]. This risk is mitigated by observing the ban on feeding swill to pigs as foreseen by the EU legislation [43]. A proper implementation of the existing rules should be ensured and a communication campaign should be addressed to pig owners to make them understand the danger of that practice. In the absence of specific legislation and in areas in which swill feeding is practiced, treatment of swill at temperatures higher than 70 °C should be guaranteed [16, 19].

##### Vehicles and other fomites, clothing, footwear, surgical equipment

African swine fever virus has a remarkable ability to survive in the environment for several days, especially if protected by organic material. The resistance of the virus to inactivation means that transmission is possible via contaminated clothing and shoes, equipment and vehicles.

Drivers and their vehicles transporting pigs to pig holdings, market or slaughterhouse, delivering feed, or collecting carcasses represent a major risk for the transmission of the disease. Vehicles used for transport of pigs shall be cleaned and disinfected immediately after every transport of animals, and if necessary before any new loading of animals using disinfectants officially authorized in cleaning and disinfection facilities approved by the competent authority, and provide documentary evidence that these operations have been performed. In cleaning and disinfecting the vehicles, special attention should be given to the truck body, the loading ramp, the equipment having been in contact with pigs, the driver’s cabin and the protective clothes/boots used during unloading. Vehicles used for transport of animals shall keep a register containing place and date of disinfection [42]. Drivers should strictly follow farm biosecurity protocols when handling animals and, as a rule, they should not enter into pig holdings, at least the area where the animals are kept. Pig keepers should take precautions against contamination from vehicles by establishing a pig loading area and by not allowing drivers into pig area [8].

Sharing of equipment between holdings should be avoided. All instruments, equipment or tools that are likely to be in contact with pigs, also the ones to restrain animals, shall be assigned to the farm and kept clean. In case they have to be transported to other farms they have to be cleaned and disinfected, also when re-introduced into the farm [8].

##### Workers and visitors

Visitors should be discouraged to enter pig holdings, both commercial and backyard. The role of people in transmitting pathogens is well documented. Appropriate hygiene measures have to be adopted by all persons entering into contact with pigs (domestic and/or feral). They can carry pathogens on clothing, footwear and hands. People entering the farm, including farmers and workers should not have been in contact with other pigs recently. In case they have, they should not be admitted to the farm.

Visitors, including farm workers, should be provided with specific clothes and footwear to be used and left into the farm. Workers, working with the herd should not have contact with other pigs and they shall not have pigs at their home. Pig workers must be well informed of their potential role in the spread of the disease.

On small farms, farmers shall avoid visiting other farms and also avoid allowing people to enter the premise. Farmers should have clothes and footwear to be worn and used when entering the pig stable [8].

##### Slurry

Faeces from ASFV infected pigs contains large amount of virus [44]. Therefore, disposal of manure, bedding material and slurry have to be considered in areas at risk. Indeed, the dispersion of pig slurry on agricultural lands is a dangerous practice, the virus can be introduced into the environment infecting wild boar and free ranging pigs. The survival of ASFV in the environment depends also on the season, cold temperature facilitates the survival of the virus whereas sunlight and drying reduce its survival. Commercial pig holdings are normally provided with storage basins, which allow manure treatment with specific disinfectants [8, 16, 45].

##### Genetic materials

There is not reliable evidence for ASFV transmission from sows to foetuses during pregnancy [46] as well as for sexual transmission in pigs. However, ASFV is shed in genital secretions and the virus was found in the semen of one boar experimentally infected [47]. Due to that, to ensure that semen is free from ASFV provisions and requirements are reported in the EU legislation [48] and by the OIE [49].

##### Bites from ticks

*Ornithodoros* spp. ticks have been found infesting pig pens in Africa and Iberian Peninsula. As described previously, such ticks can maintain ASFV infection for several months or even years after feeding on viraemic animals and, at local level, they can be involved in the maintenance and long-term transmission of ASFV. In Madagascar, ASFV was isolated from ticks found on a farm where no pigs had been introduced for at least 4 years [50]. In Portugal an outbreak was determined by the presence of ticks that harboured the virus for more than 5 years [30]. Due to the ticks long life and ability to survive for a long time without feeding, eradication of ticks from the old pigsties is invariably unsuccessful. Therefore, in order to avoid contacts, pigs shall not be housed in infested premises. The premises should be isolated with fences to prevent the access of pigs or destroyed and then new premises rebuilt in another location.

In commercial pig holdings, the above-mentioned preventive measures can be adopted with different degrees of implementation. The level of implementation varies according to the size and profile of the holding, the existent infrastructure, the epidemiological situation and the capacity of the producer to invest resources [8].

In situation of risk, the farms bio-security plans have to be verified by veterinary services and because of the virus’s ability to survive in the environment, the plans shall also include detailed procedures on cleaning and disinfection, which are an essential part of the farm biosecurity system. Lipidic solvents, which destroy the envelope of the virus and commercial disinfectants based on iodine and phenolic compounds, are effective in inactivating the ASFV [16, 19]. However, disinfectants should be officially authorized by the veterinary service and the conditions for their use strictly respected.

The situation is rather different for backyard holdings and for free-range pigs where, normally, the investment in infrastructures is minimal and few arrangements are made to provide the pigs with housing.

#### Preventive measures in backyard holdings

Backyard holdings with poor bio-security in place are currently playing an important role in the spread of ASFV in the Eastern part of Europe. Indeed, in this sector of the pig production system feeding pigs with kitchen waste is common practice and the main biosecurity measures are not easy to implement, due to the minimal investment in infrastructure typical for this type of pig production system. However, there is a set of basic preventive measures applicable also in backyard holdings and if they are properly and strictly implemented they are effective in minimizing the risk of ASFV spread. Such measures include:

- No swill feeding.
- Avoid feeding pigs with fresh fodder harvested in areas at risk for ASFV exposure.
- Buying pigs from trusted and certified sources (ASFV-free commercial holdings).
- Keeping pigs confined in stables.
- Restricting the access to pig‘s stable only to people in charge of taking care of the animals.
- People working in contact with pigs should wear clothes and footwear to be worn and used only when working in the stable and to be left in the stable after use.
- Workers cannot bring food onto the premise.
- People working in contact with pigs should wash hands with soap before entering and leaving the premise.
- Using effective disinfectants to be placed at the entrance of the stable.
- Proper disposal of dead animals or parts of dead animals to avoid the spread of infected material and also to attract wild animals.
- No wild boar or part of it, shall be brought onto the premise.
- In areas at risk for the presence of ASFV in wild boar, where the viral contamination of the environment can be high, effective disinfectants, such as calcium hydrate (slaked lime), should be spread and renewed, around pig‘s stable and at the entrance [51].
- Pig’s owner and people in charge of pigs shall avoid visiting other farms.

Furthermore, to enhance ASFV early detection:

- Veterinary services should be informed in case of dead or sick pigs.
- Home slaughtering should be carried out under veterinary supervision.

#### Preventive measures in outdoor keeping practice and free-range pigs

Biosecurity in the free ranging system is almost impossible to apply, especially in endemic areas where common pasture is practiced (i.e. municipal pastures in Sardinia or in public forests as in Georgia/Armenia). Free ranging pigs is considered a dangerous practice for the persistence of ASFV in certain areas of endemicity. Indeed, animals belonging to different herds can share the same grazing areas, they can also comingle with wild boar facilitating the transmission of the virus [6]. In such circumstances, free ranging pigs should be forbidden.

In areas at risk, biosecurity in outdoor keeping system shall be focused on segregation and feed control. Fencing or double fencing is very difficult to implement, and also to maintain, but it is the only possible mean to try to mitigate the risk of pigs direct contact with infected animals and contaminated pasture.

#### Preventive measures during hunting

Hunting is allowed in the majority of the forested areas of North-East Europe and wild boar are one of the more intensively hunted ungulate species in Europe. Yet, wild boar has expanded throughout Europe during the last 40 years [52]. Indeed, they have a high reproductive rate, and populations can double in size after each reproduction season [53]. Wild boar are susceptible to ASFV and they show similar clinical signs and mortality to domestic pigs [12, 13] with similar virus elimination timing and concentration [24]. When wild boar die, infected carcasses, if not promptly removed, remain in the environment and they can, directly or indirectly, infect other susceptible pigs, continuing the epidemiological cycle of the disease. Wild boar can also contribute to spread the virus during the infectious period of the disease, since they eliminate the virus into the environment throughout their excretions and secretions [12, 24]. In 2015, in Poland and in the Baltic countries, 897 wild boar positive for ASFV have been hunted or found dead in the affected territories (data available at: http://www.efsa.europa.eu/it/events/event/151123). Most probably the reported figure is strongly underestimated, since it appears that only 10% of the positive dead wild boars are detected in the forest [4]. Considering that a large amount of ASFV is shed during the infectious period of the disease and that the virus is rather resistant in the environment, especially if protected by organic material, it can be expected that in the affected forests, the viral contamination of the environment to be rather high. Indeed, hunting wild boar implies blood contamination of the soil, transportation of dead animals to the dressing facility (when dressing is not performed directly on the field), dressing animals, offal discharge, meat dissection and its conservation. Therefore, in the light of a control strategy, the role of the wild boar and hunting cannot be underestimated and basic preventive measures are to be adopted to minimize the risk of spread of the disease.

Minimum biosecurity requirements to apply during hunting in the affected territories:

- Hunters shall be authorised to hunt in the area only after a specific training on basic hygiene and biosecurity practices;
- Hunted wild boar should never leave the hunting area unless checked and tested and the carcasses released only when resulted negative to ASFV.
- Transport of hunted animals to the dressing facility is carried out using dedicated vehicles. Private cars shall be parked outside the hunting house, possibly on the main road.
- The use of the dressing facilities should be authorised only in case it is available: tap water, electricity, waste water collection and freezers.
- Animal dressing should be performed using appropriate aprons which must remain in the facility. Working tools cannot be transported to other places.
- Hunting suits, including boots/shoes should be kept in specific bags. Boots are worn in the dressing room before hunting and re-placed in the same bag after hunting.
- Boots and apron shall be cleaned and disinfected after each use.
- Dressing rooms are to be equipped with effective disinfectants.
- Offals should be stored in proper containers inside the dressing areas and before storing, containers shall be cleaned and sprayed with effective disinfectants.
- Ground pits for offal disposal should be at least 1.5 m deep, fenced and closed with a locked closure. Pits should be located in close proximity to the dressing room.
- Wild boar carcasses shall be individually identified before storing. In case of ASFV positive outcome all stored carcasses have to be disposed under veterinary supervision and the whole dressing room cleaned and disinfected.

## Conclusions

The current ASF situation in the East part of the EU is representing a constant threat to the EU livestock sector and the recent expansion of the disease has also demonstrated the ability of the virus to spread long distance. ASF is a disease for which there is no effective vaccine and its control relies on early detection followed by rapid eradication. Considering the epidemiological situation and the possible economic consequences of the introduction of ASF, it is extremely important that free territories are maintained free by preventing the introduction of the disease. For such purpose, bio security plays a key role in preventing ASF, and given its epidemiological cycle, simple measures may prove effective in mitigating the transmission pathways of the disease, also in the backyard sector. In certain countries, in case of occurrence of epidemic diseases, there is the attempt to try to decrease the local risk of further spread, by reducing the number of backyards, especially in the areas surrounding commercial holdings, and afterwards prohibiting this type of farming practice. Given the socio-economic relevance of the backyard sector, such discriminatory approach needs to be carefully evaluated since it might lead to poor compliance of the measures enforced to control the disease.

The final responsibility of controlling ASF belongs to the veterinary authorities. However, in risk areas, pig producers have to understand the risk posed by the presence of the disease and they have to adopt all the necessary precautionary measures to protect their own herds. To achieve this, veterinary services shall provide basic information to pig holders through appropriate communication campaigns and by promoting the adoption of preventive measures. The key for changing behaviours and practices lies in people’s perception of the level of risk and in such situation, it is of crucial importance that all the elements of the pig production and marketing chain, including backyards, transporters, service providers and slaughterhouses, are involved in the implementation of the control strategy for the disease.

In adopting an eradication strategy against ASF, the role of wild boar cannot be underestimated and preventive measures need to be established also to control the possible pathways of ASFV transmission from wild to domestic pigs. Hunters may have an active role in preventing the spread of ASFV and they should be involved in the disease control strategy since the beginning.
